# Supplementary material for: Mental health among healthcare workers and other vulnerable groups during the COVID-19 pandemic and other coronavirus outbreaks: A rapid systematic review
Source: PLoS One. 2021 Aug 4;16(8):e0254821. doi: 10.1371/journal.pone.0254821 (PMC8336853; doi:10.1371/journal.pone.0254821)
Supplement: S2 Appendix — (DOCX) [file pone.0254821.s002.docx]

# Appendix

Search strategies

Summary

MEDLINE (Sets 1-3) n=207 [Email of 08/07/2020] [Reviews]

MEDLINE (Set 4) n=1031 [Email of 08/07/2020] [Studies]

MEDLINE (Set 5) n=306 [Email of 09/07/2020] [Studies]

PsyRXiv n=142 [Email of 22/07/2020] [Reviews]

MedRxiv n=51 [Email of 11/08/2020] [Reviews]

CORD-19 Dataset n= 471+6 [Emails of 10/08/2020] [Reviews]

Total=2214

Database: Ovid MEDLINE(R) and Epub Ahead of Print, In-Process & Other Non-Indexed Citations and Daily <1946 to July 06, 2020>

Search Strategy

--------------------------------------------------------------------------------

[COVID-19]

1     coronaviridae infections/ or exp coronavirus infections/ (20419)

2     (coronaviridae or coronavirus or betacoronavir* or beta-coronavir* or nCoV* or COVID-19* or COVID19* or COVID-2019* or COVID2019* or nCoV* or 2019nCoV*).ti,ab,kf. or (coronavir* or covid*).ti. (40339)

3     (severe acute respiratory syndrome or SARS or Middle East Respiratory Syndrome or MERS).ti,ab,kf. (23421)

4     (severe acute respiratory syndrome or Middle East Respiratory Syndrome).hw. (5738)

5     (lockdown or lock down*).mp. (902)

6     ((epidemic? or pandemic* or global* or international or worldwide or world wide) adj5 (quarantine? or isolat*)).mp. (5555)

7     disease outbreaks/px (3)

8     or/1-7 (59029)

[Systematic Review Filter]

9     (systematic or structured or evidence or trials or studies).ti. and ((review or overview or look or examination or update* or summary).ti. or review.pt.) (212521)

10     (0266-4623 or 1469-493X or 1366-5278 or 1530-440X or 2046-4053).is. (18817)

11     meta-analysis.pt. or (meta-analys* or meta analys* or metaanalys* or meta synth* or meta-synth* or metasynth*).ti,ab,kf,hw. (205807)

12     ((systematic or meta) adj2 (analys* or review)).ti,kf. or ((systematic* or quantitativ* or methodologic*) adj5 (review* or overview*)).ti,ab,kf,sh. or (quantitativ$ adj5 synthesis$).ti,ab,kf,hw. (273552)

13     (integrative research review* or research integration).tw. or scoping review?.ti,kf. or (review.ti,kf,pt. and (trials as topic or studies as topic).hw.) or (evidence adj3 review*).ti,ab,kf. (195557)

14     review.pt. and ((medline or medlars or embase or pubmed or scisearch or psychinfo or psycinfo or psychlit or psyclit or cinahl or electronic database* or bibliographic database* or computeri#ed database* or online database* or pooling or pooled or mantel haenszel or peto or dersimonian or der simonian or fixed effect or ((hand adj2 search*) or (manual* adj2 search*))).tw,hw. or (retraction of publication or retracted publication).pt.) (154311)

15     (rapid review? or (mixed method? adj (synthes* or research or review)) or (thematic adj (review or synthes* or summary)) or ((integrative or realist) adj (synthes* or review)) or (narrative adj (review or synthes* or summary))).mp. (20101)

16     or/9-15 (576345)

17     8 and 16 (1484)

[Health Disparities/Characteristics]

18     Residence Characteristics/ or Environment design/ or (neighbo?rhood* or residential environment*).mp. (62332)

19     (home ownership or (housing adj (instabilit* or insecurit* or stabilit* or strain or securit*)) or (mortgage adj (arrear? or debt? or default* or delinquency or problem* or strain)) or (foreclosure or eviction) or (housing adj (loss or repossession*)) or (repossess* adj3 (hous* or propert*))).mp. (2012)

20     exp Marital status/ or (marital status or marriage status or widow* or cohabit* or divorce* or single parent* or live* alone).mp. (69285)

21     (rural* or innercity or innercities or (inner adj (city or cities)) or (household adj2 size) or overcrowding).mp. (178415)

22     (homeless* or (living adj1 (outside or inside or near* or adjacent))).mp. (16482)

23     or/18-22 (313600)

24     Acculturation/ or Cross-Cultural Comparison/ or Culture/ or Cultural Characteristics/ or Cultural Deprivation/ or Cultural Diversity/ (87313)

25     exp Continental Population Groups/ or exp "Emigrants and Immigrants"/ or exp Ethnic Groups/ or "Transients and Migrants"/ or Minority groups/ or Minority health/ or Prejudice/ or exp Race Relations/ or Racism/ or Refugees/ or Social Discrimination/ or Xenophobia/ (347466)

26     (ethnic* or ethnology or minorit* or migration background or race or racial or racism).mp. (430366)

27     Language.sh. or (nonEnglish or non-English or english as a second language or foreign language or language other than or aboriginal or first nation* or eskimo or hispanic* or indigenous or inuit or latino* or latina* or native american or whites or caucasian* or nonwhite* or non-white* or Torres Strait Islander).mp. (234935)

28     or/24-27 (758633)

29     (unemployment or unemployment).mp. (14216)

30     exp Gender Identity/ or Women's Health/ or Sex Factors/ (306363)

31     (gender differences or gender identity or gender* role? or men? role? or man? role? or sex disparit* or sex difference? or sex role or wom#n? role?).mp. (84388)

32     30 or 31 (348064)

33     exp Educational status/ or Education/ (71793)

34     ((education* adj2 level?) or educational status or ((higher or better or worse or less) adj educated) or ((higher or better or worse or less) adj level? of education) or schooling).mp. (101996)

35     33 or 34 (121931)

36     religi*.mp. (62790)

37     Hierarchy, Social/ or Psychosocial Deprivation/ or Social determinants of Health/ or Sociological Factors/ or Working Poor/ (7847)

38     (concentration index or deprivation or disparit* or equity or gini or inequalit* or inequit*).mp. (214854)

39     Income/ or exp Poverty/ or Socioeconomic Factors/ or exp Social Class/ or Social Welfare/ (246645)

40     ((social adj (circumstance? or background or class* or determinant? or position or status)) or SES or sociodemographic? or socio-demographic? or socio-economic? or socioeconomic?).mp. (334053)

41     (assets index or disadvantaged or economic level or impoverished or income? or poverty).mp. (192727)

42     or/37-41 (635247)

43     Anomie/ or Social Capital/ or exp Social Environment/ or Social Conditions/ or Social Control, Informal/ or Social Isolation/ or Social Marginalization/ or Social Participation/ or Social Stigma/ or exp Social Support/ or Trust/ (157696)

44     (anomie or civil society or (community adj3 (capital or cohes* or participa*)) or (emotional or (psychosocial adj support)) or ((neighbourhood or neighborhood) adj cohes*) or (social adj (capital or cohes* or exclusion or influence? or network* or organis* or organiz* or participation or relationships or support)) or collective efficacy or informal social control or (neighbo?rhood adj (cohesion or disorder)) or (soci* adj context*) or soci*-context* or social disorgani?ation or trust).mp. (342200)

45     43 or 44 (407937)

46     Health Equity/ or Health Services Accessibility/ or Health Status Disparities/ (88913)

47     (healthcare disparit* or health care disparit* or health status disparit* or health disparit* or health inequalit* or health inequit* or medically underserved).mp. (52111)

48     46 or 47 (118485)

49     23 or 28 or 29 or 32 or 35 or 36 or 42 or 45 or 48 (2122409)

50     (disadvant* or discriminat* or disparit* or diversit* or equal* or equit* or inequ*).ti. (140694)

51     ((health* or social* or racial* or cultural* or gender*) adj (disadvant* or discriminat* or disparit* or diversit* or equal* or equit* or inequ*)).ti,ab,kf. (44898)

52     ((depriv* or underserv* or un-deserv*) adj2 (communit* or neighbourhood? or population?)).ti,ab,kf. (6112)

53     (difficult* adj2 (accessing or engag* or empower* or participat* or reach or outreach or out-reach)).ti,ab,kf. (3974)

54     ("hard to reach" or "hard to access").ti,ab. (2150)

55     (engagement or disengagement or dis-engagement).ti,kf. (14529)

56     (intersectionalit* or inter-sectionalit*).ti,ab,kf. (877)

57     (protected characteristic? or enhanced protection).ti,ab,kf. (871)

58     or/49-57 (2225897)

59     17 and 58 (111)

[Mental Health]

60     mental disorders/ or anxiety disorders/ or agoraphobia/ or anxiety, separation/ or neurocirculatory asthenia/ or neurotic disorders/ or obsessive-compulsive disorder/ or hoarding disorder/ or panic disorder/ or phobic disorders/ or phobia, social/ or "bipolar and related disorders"/ or bipolar disorder/ or trichotillomania/ or dissociative disorders/ or multiple personality disorder/ or "feeding and eating disorders"/ or anorexia nervosa/ or binge-eating disorder/ or bulimia nervosa/ or mood disorders/ or depressive disorder/ or depression, postpartum/ or depressive disorder, major/ or depressive disorder, treatment-resistant/ or dysthymic disorder/ or premenstrual dysphoric disorder/ or seasonal affective disorder/ or cyclothymic disorder/ or mutism/ or reactive attachment disorder/ or "fetishism (psychiatric)"/ or personality disorders/ or antisocial personality disorder/ or borderline personality disorder/ or compulsive personality disorder/ or dependent personality disorder/ or histrionic personality disorder/ or hysteria/ or paranoid personality disorder/ or passive-aggressive personality disorder/ or schizoid personality disorder/ or schizotypal personality disorder/ or "schizophrenia spectrum and other psychotic disorders"/ or affective disorders, psychotic/ or capgras syndrome/ or delusional parasitosis/ or paranoid disorders/ or psychotic disorders/ or psychoses, substance-induced/ or psychoses, alcoholic/ or schizophrenia/ or schizophrenia, catatonic/ or schizophrenia, disorganized/ or schizophrenia, paranoid/ or shared paranoid disorder/ or sexual dysfunctions, psychological/ or somatoform disorders/ or body dysmorphic disorders/ or conversion disorder/ or hypochondriasis/ or munchausen syndrome/ or munchausen syndrome by proxy/ or neurasthenia/ or "trauma and stressor related disorders"/ or adjustment disorders/ or stress disorders, traumatic/ or combat disorders/ or psychological trauma/ or stress disorders, post-traumatic/ or stress disorders, traumatic, acute/ (576834)

61     catatonia/ or delusions/ or depersonalization/ or depression/ or malingering/ or obsessive behavior/ or stalking/ or paranoid behavior/ or schizophrenic language/ or self-injurious behavior/ or self mutilation/ or suicide/ or suicidal ideation/ or suicide, attempted/ or impulsive behavior/ or compulsive behavior/ or behavior, addictive/ or hoarding/ (211488)

62     Emotions/ or Emotional Adjustment/ or emotion*.ti,ab. (218956)

63     (bereavement or grief).mp. (17491)

64     (stigma* or prejudice).mp. (63161)

65     Mental Health/ or ((mental* adj2 (disorder* or health* or well* or ill*)) or wellbeing or well-being).ti,ab,kf. (305669)

66     mental health services/ or community mental health services/ or community psychiatry/ or (counselling or counseling).mp. (171082)

67     (acute stress or adjustment disorder* or ADNOS or affective disorder* or agoraphobi* or anorexia nervosa or anxiety or astheni* or attachment disorder* or BPD or binge eat* or binging or bipolar or body dysmorphi* or bulimi* or catatoni* or combat disorder* or compulsi* or conversion disorder* or cyclothymi* or delusion* or depersonali#ation or depressed or depression or depressive or dissociative disorder* or dyssomni* or dyspareunia* or dysphori* or dysthymi* or dystoni* or eating disorder* or EDNOS or emotional trauma or fear or health anxiety or hoarding or hyperactivity or hypochondri* or hysteri* or medically unexplained or malingering or mania or manic or MDD or mental or mood? or munchausen or MUPS or mutism or neurastheni* or neurotic or neuros* or obsess* or panic or paranoi* or parasuicid* or perceptual disorder* or personality disorder* or phobi* or PND or ((post-trauma* or posttrauma*) adj stress*) or psychiatr* or psychogenic or psychopathol* or psychosomatic or psychotic or psychos* or PTSD or schizo* or (self adj (injur* or harm or mutilat*)) or (sexual dysfunction* adj3 psycho*) or social anxiety or somati* or somatoform or suicid* or trichotillomani* or stalking).ti,ab,kf,kw. (1685729)

68     Adaptation, Psychological/ or Stress, Psychological/ (196502)

69     (psychological adj3 (health* or stress* or well* or impact? or outcome? or advers* or protecti*)).mp. (153466)

70     (mental* or psychological or psychosocial*).ti. (193234)

71     psychology.fs. (1058758)

72     or/60-71 (2696315)

73     17 and 72 (125)

74     SOCIAL ISOLATION/ (13260)

75     *loneliness/ or social alienation/ (3552)

76     (loneliness or social inclusion or social participation).ti,kf. or (communit* and (social adj (isolation or participation))).mp. (7013)

77     ((subjective or objective) adj social isolation).ti,ab,kf. (43)

78     ((chang* or develop* or enhanc* or initiative? or intervention? or program* or mitigat* or address* or improv* or target*) adj5 (loneliness or ((social or community) adj (connect* or inclusion or isolation or network? or participation or relations*)))).ti,ab,kf. (5789)

79     ((alleviat* or ease or manag* or mitigat* or prevent* or overcom* or reduc* or stop*) adj5 (social* or communit*) adj5 (alienat* or discriminat* or excluded or exclusion or isolated or isolation)).ti,ab,kf. (970)

80     ((alleviat* or ease or manag* or mitigat* or prevent* or overcom* or reduc* or stop*) adj2 (isolation or isolated or exclusion or excluded)).ti,ab,kw. (4688)

81     ((address* or enhanc* or improv* or increas* or promot* or target*) adj2 (inclusion or inclusivity)).ti,ab,kf. (2582)

82     ((address* or enhanc* or improv* or increas* or promot* or target*) adj3 (social* or communit*) adj3 network?).ti,ab,kf. (745)

83     social participation/ (2390)

84     ((social or psychosocial) adj support*).ti,kf. (11760)

85     "sense of belonging".ti,ab,kf. (963)

86     (separation adj1 anxiety).mp. (3068)

87     or/74-86 (47880)

88     17 and 87 (17)

89     **59 or 73 or 88 (207)**

90     8 and 58 (3823)

91 8 and 72 (3899)

92     8 and 87 (323)

93 90 or 91 or 92 (6863)

94 **90 and (91 or 92) (1031)**

95 ((frontline or front-line or health* or healthcare*) adj2 (worker* or staff? or employee* or doctor* or medic? or clinician? or surgeon? or nurs*)).mp.

96 **(8 and (72 or 87) and 95) (+306)**

-------------------------------------------------------------

**PsyArXiv**

#1 (review OR overview OR meta-analys* OR metaanalys* OR meta-synth* OR metasynth* OR (meta* AND analys*) OR (meta* AND synth*) OR (quantitative AND synthes*) OR (mixed AND method*) OR (research AND integration) OR systematic OR thematic OR realist OR narrative OR retraction): 1840 results

AND

#2 (COVID OR COVID-19 OR COVID19 OR COVID-2019 OR COVID2019 OR nCoV OR nCoV19 OR nCoV-19 OR nCoV2019 OR nCoV-2019 OR 2019nCoV OR 2019-nCoV OR "covid 19" OR "covid 2019" OR coronaviridae OR coronavirus OR betacoronaviridae  OR betacoronavirus OR beta-coronaviridae OR beta-coronavirus OR "severe acute respiratory syndrome" OR SARS OR "Middle East Respiratory Syndrome" OR MERS OR epidemic OR epidemics OR pandemic OR pandemics OR lockdown OR lock-down OR "locked down"): 989 results

#3 (#1 and #2) 142 results

-------------------------------------------------------------

**MedRxiv**

via <https://mcguinlu.shinyapps.io/medrxivr/>

[Cc][Oo][Vv][Ii][Dd]

[Cc]oronavir

[Nn][Cc][Oo][Vv]

[Ss]evere [Aa]cute [Rr]espiratory [Ss]yndrome

SARS

[Mm]iddle [Ee]ast [Rr]espiratory [Ss]yndrome

MERS

[Ll]ockdown

[Ll]ock-down

[Ll]ocked down

[Ee]pidemic

[Ee]pidemics

[Pp]andemic

[Pp]andemics

[Qq]uarantine

[Aa]nxiety disorder

[Aa]nxiety disorders

[Aa]cute stress

[Aa]djustment disorder

ADNOS

[Aa]ffective disorder

[Aa]ffective disorders

[Aa]goraphobia

[Aa]norexia

[Aa]sthenia

[Aa]ttachment disorder

[Aa]ttachment disorders

BPD

[Bb]inge eat

[Bb]inge eating

[Bb]inging

[Bb]ipolar

[Bb]body dysmorphia

[Bb]ulimia

[Bb]urnout

[Bb]urn-out

[Cc]atatonia

[Cc]ombat disorder

[Oo]bsessive compulsive disorder

[Cc]onversion disorder

[Cc]yclothymia

[Dd]elusions

[Dd]elusional

[Dd]epersonalization

[Dd]epersonalisation

[Dd]epressed

[Dd]epressive

[Dd]epressives

[Dd]epression

[Dd]issociative disorder

[Dd]yssomnia

[Dd]yspareunia

[Dd]ysphoria

[Dd]ysthymia

[Dd]ystonia

[Ee]ating disorder

[Ee]ating disorders

EDNOS

[Ee]motional

[Ff]ear

[Hh]ealth anxiety

[Hh]oarding

[Hh]yperactivity

[Hh]ypochondria

[Hh]ysteria

[Ll]oneliness

[Mm]edically unexplained

[Mm]alingering

[Mm]ania

[Mm]anic

MDD

[Mm]ental

[Mm]ood

[Mm]oods

[Mm]unchausen

MUPS

[Mm]utism

[Nn]eurasthenia

[Nn]eurotic

[Nn]euroses

[Nn]eurosis

[Pp]anic

[Pp]aranoid

[Pp]arasuicide

[Pp]arasuicides

[Pp]erceptual disorder

[Pp]ersonality disorder

[Pp]hobic

[Pp]hobia

[Pp]hobias

PND

[Pp]ost-trauma

[Pp]osttrauma

[Pp]ost-traumatic

[Pp]osttraumatic

PTSD

[Ss]tress disorder

[Ss]tress disorders

[Pp]sychiatry

[Pp]sychiatric

[Pp]sychological

[Pp]sychosis

[Pp]sychoses

[Ss]chizoaffective

[Ss]elf-harm

[Ss]elfharm

[Mm]utilate

[Mm]utilation

[Ss]ocial anxiety

[Ss]omatoform

[Ss]uicide

[Ss]uicides

[Ss]uicidal

[Tt]richotillomania

[Ss]talking

[Pp]sychosocial

[Ss]ystematic [Rr]eview

[Mm]eta-analysis

[Mm]etaanalysis

[Tt]hematic [Rr]eview

[Rr]ealist [Rr]eview

[Nn]arrative [Rr]eview

[Rr]apid [Rr]eview

[Oo]verview of [Rr]eviews

[Uu]mbrella [Rr]eview

RESULTS (51)

-------------------------------------------------------------

**CORD-19 Dataset**

Methods.

CORD-19 dataset in Rayyan (8-Aug-2020), n=210,537 records.

Exported from Rayyan (enw format) & imported into EndNote

Filter-1 Title=*review or meta-analysis or systematic,*n=9936

Filter-2 Title words ‘contain’ 81 terms for CMD as listed below

or Filter-3 Title words searched for *Covid* and any field=*mental health*

CMD Terms (and word stems)

psychological, anxiety, acute stress, adjustment disorder, ADNOS, affective disorder, agoraphobi, anorexia nervosa, anxiety, astheni, attachment disorder*, BPD, binge eat, binging, bipolar, body dysmorphi, bulimi, burnout, catatoni, combat disorder, compulsi, conversion disorder, cyclothymi, delusion, depersonali, depress [depressed, depression, depressive], dissociative disorder, dyssomnia, dyspareunia, dysphori, dysthymi, dystoni, eating disorder, EDNOS, emotional, fear, health anxiety, hoarding, hyperactivity, hypochondri, hysteri, loneliness, medically unexplained, malingering, mania, manic, MDD, mental, mood, munchausen, MUPS, mutism, neurastheni, neurotic, neuroses, neurosis, obsess, panic, paranoi, parasuicide, perceptual disorder, personality disorder, phobi, PND, post-trauma, posttrauma, stress disorder, psychiatry, psycho, PTSD, schizo, self-harm, mutilat, social anxiety, somati, somatoform, suicide, trichotillomania, stalking, social, psychosocial

Total=477

**************************************************************************
