# Supplementary material for: Mental health among healthcare workers and other vulnerable groups during the COVID-19 pandemic and other coronavirus outbreaks: A rapid systematic review
Source: PLoS One. 2021 Aug 4;16(8):e0254821. doi: 10.1371/journal.pone.0254821 (PMC8336853; doi:10.1371/journal.pone.0254821)
Supplement: S3 Appendix — (DOCX) [file pone.0254821.s003.docx]

INCLUDED SYSTEMATIC REVIEWS NOT CITED

1. De Brier N, Stroobants S, Vandekerckhove P, De Buck E. Factors affecting mental health of health care workers during coronavirus disease outbreaks: a rapid systematic review. PsyArXiv. 2020. Doi: <https://doi.org/psyarxiv.com/w9uxsPsyArXiv>.
2. Ricci Cabello I, Meneses Echavez JF, Serrano-Ripoll MJ, Fraile-Navarro D, Fiol de Roque MA, Moreno GP, et al. Impact of viral epidemic outbreaks on mental health of healthcare workers: a rapid systematic review. MedRXiv. 2020. Doi: https://doi.org/10.1101/2020.04.02.20048892.
3. Muller AE, Hafstad EV, Himmels JPW, Smedslund G, Flottorp S, Stensland SØ,et al. The mental health impact of the covid-19 pandemic on healthcare workers, and interventions to help them: a rapid systematic review. Psych Res. 2020;293:113441. Doi: <https://doi.org/10.1016/j.psychres.2020.113441>.
4. Mahbub H, Sultana A, Purohit M. Mental health outcomes of quarantine and isolation for infection prevention: a systematic umbrella review of the global evidence. EpiH. 2020;42:e2020038. Doi: <https://doi.org/10.4178/epih.e2020038>.
5. Kisely S, Warren N, McMahon L, Dalais C, Henry I, Siskind E. Occurrence, prevention, and management of the psychological effects of emerging virus outbreaks on healthcare workers: rapid review and meta-analysis. BMJ. 2020;369:m1642. Doi: <https://doi.org/10.1136/bmj.m1642>.
6. Pappa S, Ntella V, Giannakas T, Giannakoulis VG, Papoutsi E, Katsaounou P. Prevalence of depression, anxiety, and insomnia among healthcare workers during the COVID-19 pandemic: A systematic review and meta-analysis. Brain Behav Immun. 2020;08:08. Doi: <https://doi.org/10.1016/j.bbi.2020.05.026>..
7. Kisely S, Warren N, McMahon L, Dalais C, Henry I, Siskind E. Occurrence, prevention, and management of the psychological effects of emerging virus outbreaks on healthcare workers: rapid review and meta-analysis. BMJ. 2020;369:m1642. Doi: <https://doi.org/10.1136/bmj.m1642>.
8. Pappa S, Ntella V, Giannakas T, Giannakoulis VG, Papoutsi E, Katsaounou P. Prevalence of depression, anxiety, and insomnia among healthcare workers during the COVID-19 pandemic: A systematic review and meta-analysis. Brain Behav Immun. 2020;08:08. Doi: <https://doi.org/10.1016/j.bbi.2020.05.026>.
